# Pathology and Treatment of Traumatic Cervical Spine Syndrome: Whiplash Injury

**DOI:** 10.1155/2018/4765050

**Published:** 2018-02-28

**Authors:** Nobuhiro Tanaka, Kivanc Atesok, Kazuyoshi Nakanishi, Naosuke Kamei, Toshio Nakamae, Shinji Kotaka, Nobuo Adachi

**Affiliations:** ^1^Department of Orthopaedic Surgery, Institute of Biomedical & Health Sciences, Hiroshima University, Hiroshima, Japan; ^2^Department of Orthopaedic Surgery, University of Alabama at Birmingham, Birmingham, AL, USA

## Abstract

Traumatic cervical syndrome comprises the various symptoms that occur as a result of external force such as that of a traffic accident. In 1995, the Quebec Task Force on whiplash-associated disorders (WAD) formulated the Quebec classification, with accompanying clinical practice guidelines. These guidelines were in accordance with the stated clinical isolated or combined symptoms of the syndrome: neck pain, headaches, dizziness, numbness of head or face, eye pain, vision loss, double vision, tinnitus, hearing loss, nausea, and numbness and/or weakness of extremities. In recent years, cerebrospinal fluid hypovolemia or fibromyalgia has been recognized as a major notable cause of a variety of symptoms, although many clinical questions remain regarding the pathology of this syndrome. Therefore, its diagnosis and treatment should be conducted extremely carefully. While the Quebec classification and its guidelines are very useful for the normalization and standardization of symptoms of traumatic cervical syndrome, in the future, we would like to see the emergence of new guidelines that better address the diversity of this disease.

## 1. Introduction

Traumatic cervical syndrome is defined as the “biological and neurological consequences for the cervical spine and nervous system caused by neck trauma, and is a syndrome comprising various symptoms of the motor and nervous system [1] but also mental, neurological, as well as otological and visual balance dysfunction” [2, 3] (Table 1). The symptoms can include minor neck trauma without the so-called “whip” movement of the neck. “Whiplash injury,” which has been traditionally diagnosed after a traffic injury, is pathologically incorrect and should not be used as part of clinical language, because it may cause various misunderstandings among communities and patients. Most injuries occur during a rear-end auto accident, but the injury can also result from a sports accident, physical abuse, or other trauma. Also Ferrari [4] noticed that whiplash is an example of illness induced by society, in general, and by physicians in particular. These symptoms may appear even in the absence of any visible injuries.

The report of the Quebec Task Force on whiplash-associated disorders in 1995 classified whiplash-associated disorders (WAD) into five grades based on symptoms and severity [2]. In this manuscript, we discuss the clinical signs of traumatic cervical syndrome which conform to the Quebec classification.

## 2. Quebec Classification

The Quebec classification includes neck symptoms and a range of neurological problems, as well as spine fractures or dislocations, and it is divided into 5 grades from 0 to IV [2] (Tables 2 and 3). Grades 0, I, and II correspond to the so-called “whiplash injury,” and grades III and IV are classified as traumatic cervical spinal cord injury. Symptoms such as dizziness, tinnitus, headache, memory loss, swallowing, and temporomandibular joint pain can appear in any grade.

## 3. Clinical Symptoms

### 3.1. Neck Pain

Although symptoms of traumatic cervical syndrome vary from patient to patient (Table 1), neck pain and cervical discomfort are typical symptoms [1]. Deans et al. [6] reported that neck pain occurs in 65% of patients within 6 hours, 93% within 24 hours, and 100% within 72 hours after neck injury. Many factors can influence the extent and location of the injury, such as traffic accident specifics (speed, direction, and safety equipment) and the state of the victim's cervical spine. In addition to the constructional elements of the cervical spine such as muscles, intervertebral discs, and facet joints, the injury may be caused by neural elements including the spinal dorsal root ganglion, vertebral artery, and the sympathetic nervous system. Typical clinical characteristics of the injury include the patient not complaining of neck pain immediately after the accident, but then complaining of neck pain a few hours later or the next day. This time lag can be explained by synovitis of the facet joints, where the synovial tissue involved in the facet joint has been damaged by nonphysiological behavior during a collision, which may induce synovitis of the facet joint after several hours, leading to neck pain and a limited range of motion [7].

In general, traumatic cervical spine syndrome is not protracted, and many patients recover from the symptoms within a few weeks or months. However, some reports suggest that neck pain and headaches continue for several years in 20–40% of patients, with 3-4% of patients unable to return to work [8]. Radanov et al. [9] reported that 97% of chronic traumatic cervical syndrome patients have neck pain. Clinical conditions are usually complicated and enigmatic when the injury is chronic, while the potential exists for abnormally prolonged arthritis of the synovial membrane, cervical nerve root irritation of the posterior branches, and vestibular reflex abnormalities due to vestibular dysfunction, neck muscle tension, or fibromyalgia (see below), but details of the pathology remain unclear.

### 3.2. Headache

Headaches present as chronic symptoms in 70% of patients [8]. According to the headache classification proposed by the International Headache Society (2nd edition) [10] (Section 3.2.1), a cervicogenic headache is defined as a headache resulting from disorders of the cervical spine. The diagnostic criteria following the blockade of a cervical structure include referred pain from a source in the neck, perceived in the head and/or face, and evidence of a disorder or lesion within the cervical spine or soft tissues of the neck.

**Figure 1:**
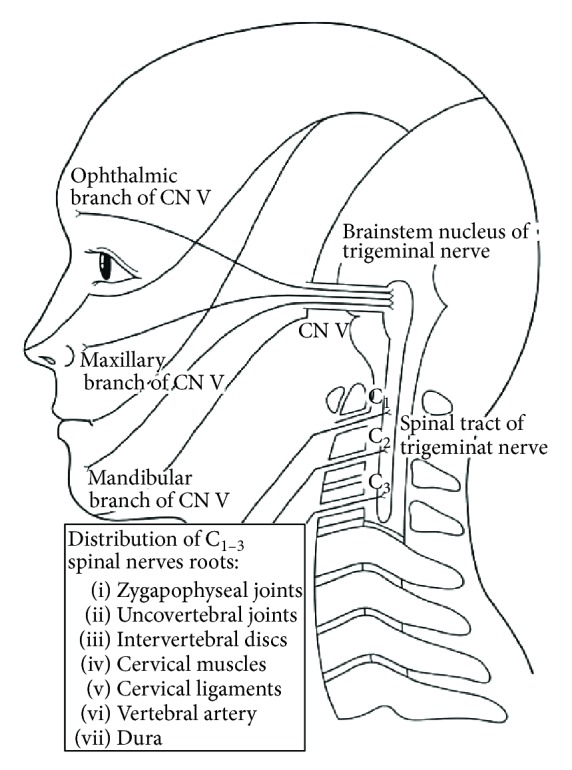
Schema of connection between upper spinal nerve roots and trigeminal nerve [5].

Some of the proposed trigger mechanisms of a cervicogenic headache are compression or inflammation of the C2 nerve root; referred pain of the first branch of the trigeminal nerve from C2 nerve root irritation trough anastomosis; emerging pain in the trigeminal nerve area caused by stimulation of the upper spinal nerve roots from the anatomical connection between the spinal trigeminal nucleus and dorsal horn at the C2-3 spinal level [5] (Figure 1); a tension headache due to entrapment of the occipital nerve after pericranial muscles constrict from cervical muscle strain or spasm. Many cervicogenic headaches are caused by movement of the neck, and pain is usually located in the occipital region, with persistent pain gradually increasing on an elapsed chronic course. Psychological factors such as fatigue, lack of sleep, stress, and depression are reported to influence cervicogenic headaches [7].

#### 3.2.1. Diagnostic Criteria of Cervicogenic Headache [8]

- Pain, referred from a source in the neck and perceived in one or more regions of the head and/or face, fulfilling criteria (C) and (D)
- Clinical, laboratory, and/or imaging evidence of a disorder or lesion within the cervical spine or soft tissues of the neck known to be, or generally accepted as, a valid cause of headache (Tumors, fractures, infections, and rheumatoid arthritis of the upper cervical spine have not been validated formally as causes of headache but are nevertheless accepted as valid causes when demonstrated to be so in individual cases. Cervical spondylosis and osteochondritis are not accepted as valid causes for the fulfillment of criterion (B). When myofascial tender spots are the cause, the headache should be coded under 2,* tension-type headache.*)
- Evidence that the pain can be attributed to the neck disorder or lesion based on at least one of the following:
  1. Demonstration of clinical signs that implicate a source of pain in the neck (Clinical signs acceptable for criterion C1 must have demonstrated reliability and validity. The future task is the identification of such reliable and valid operational tests. Clinical features such as neck pain, focal neck tenderness, history of neck trauma, mechanical exacerbation of pain, unilaterality, coexisting shoulder pain, reduced range of motion in the neck, nuchal onset, nausea, vomiting, and photophobia are not unique to a cervicogenic headache. These may be features of a cervicogenic headache, but they do not define the relationship between the disorder and the source of the headache.)
  2. Abolition of headache following diagnostic blockade of a cervical structure or its nerve supply using placebo or other adequate controls (Abolition of headache means complete relief of headache, indicated by a score of zero on a visual analogue scale (VAS). Nevertheless, acceptable as fulfilling criterion C2 is ≥90% reduction in pain to a level of <5 on a 100-point VAS.)
- Pain resolves within 3 months after successful treatment of the causative disorder or lesion

### 3.3. Vertigo

There are two types of dizziness: vertigo and planktonic, and physicians should be careful in their diagnosis of the symptoms, because brain-stem bleeding sometimes manifests itself as vertigo, when in fact it is mostly caused by an inner ear disorder. Planktonic dizziness is caused by craniocervical disorder (Chiari malformation, spinal cord tumor, etc.) or failure of the input system regarding the spinal cord. Dizziness originating from the cervical spine is collectively known as cervical vertigo, and it may be caused by circulatory failure of the vertebral artery, proprioceptor dysfunction of the cervical spine (nerve root or spinal cord disorders at C1—3), or cervical sympathetic nervous system disorders (Barré-Lieou syndrome). Hinoki [11] proposed a hypothesis in which the hypothalamus plays a fundamental role in explaining vertigo after injury. They reported that autonomic reflexes in patients with whiplash injury can be explained as being not only due to overexcitation of the cervical sympathetic nerves, but also due to the cervical and lumbar proprioceptors in producing vertigo.

### 3.4. Barré-Lieou Syndrome

Symptoms of Barré-Lieou syndrome include headache, dizziness, and other cervical sympathetic nervous system disorders, some of which can be classified as traumatic. Hypertension of the cervical sympathetic nerves and vertebral arterial circulatory disorders, stem failure, peripheral vestibular disorders, and psychogenic problems are caused mainly by stress. These symptoms are known as posterior cervical sympathetic syndrome, previously recognized as a syndrome associated with chronic cervical disease, but recently it often comprises a convenient but false diagnosis for atypical symptoms [12].

### 3.5. Numbness in Head and Face

Disruption of the trigeminal spinal nucleus, which conveys superficial facial perception along the C2-3 spinal level (see above), can lead to numbness or loss of sensation around the face in an “onion skin” distribution area, followed by a spinal cord lesion at the upper cervical spine [6].

### 3.6. Eye Symptoms

There are reports that eye symptoms emerge in 35% of traumatic cervical syndrome patients [13]. Eye pain due to trigeminal nerve stimulation, eye movement disorder (double vision) caused by oculomotor nerve disorders, visual deficit due to optic nerve disorders, and blepharoptosis due to sympathetic nervous system disorders are present.

### 3.7. Nausea and Vomiting

It is reported that nausea and vomiting are found in 17 to 29% of patients. Almost all cases have neck pain complications. Subsequently these symptoms persist for more than 6 months in 33% of patients [7].

### 3.8. Limb Symptoms

Numbness or muscle weakness of the upper extremities may appear as nerve root or spinal cord symptoms, although limb symptoms of traumatic cervical syndrome often do not produce any findings in imaging studies. Because there are few available laboratory procedures for these patients, appropriate treatment based on their pathophysiology is difficult to administer.

De Reuck [14] reported the usefulness of investigating motor evoked potentials (MEPs) in patients who had been suffering from grade II WAD for more than 6 months. They found that 13 patients had prolonged central (CMCT) and/or peripheral motor conduction times (PMCT) compared to normal values, and they recommended that MEP examination be performed in all patients with persistent pain even in the absence of objective neurological signs and nonsignificant changes on imaging. In our study, we also used electrophysiological examination using MEPs following transcranial magnetic stimulation for traumatic cervical syndrome patients, and we found significant elongation in PMCT and/or distal latencies following nerve stimulation at the cervical nerve root or Erb's point in 4 out of 11 patients who had limb symptoms without significant imaging abnormalities but with no obvious CMCT abnormalities [15]. These results may indicate potential nerve disorder in the cervical nerve root or brachial plexus in patients with traumatic cervical syndrome, even in those who develop symptoms of malaise without obvious muscle atrophy or electromyographic changes.

### 3.9. Other

The mechanisms of the following symptoms are unclear: ringing in the ears, hearing loss, insomnia, loss of concentration, fatigability, fever, memory loss, temporomandibular joint pain, and anginal-like chest pain (cervical angina).

## 4. CSF Hypovolemia

In Japan, some physicians have insisted that the cause of CSF hypovolemia is traumatic CSF leak, and they call this syndrome traumatic CSF hypovolemia [16]. CSF hypovolemia syndrome exhibits a variety of symptoms such as neck pain, visual deficit, double vision, dizziness, nausea, vomiting, ringing in the ears, hearing loss, and headaches. These symptoms resemble those of a whiplash injury, and some of them occur sporadically as traumatic cervical syndrome [17–19]. The diagnostic criteria of traumatic CSF leakage is defined as follows: (1) early vesicular radioisotope cisternography (RIC) accumulation (EVA); (2) promoted radioisotope (RI) clearance from the spinal cavity; (3) presence of abnormal paraspinal RI accumulation (PSA) [20]. However, Hashizume et al. [21] reported that traumatic CSF leak was not observed on CT myelography findings in patients with WAD, in whom CSF leak was suspected when comparing the RIC. An epidural blood patch (EBP) is the therapy of choice in patients with chronic WAD with a suspected CSF leak. Treatment for traumatic cervical spine syndrome should be uniform and logically based on medical science, and invasive treatment such as EBP should be carefully performed only in selected patients. The association of a CSF leak with chronic WAD has never been established, although its symptoms may be reduced following treatment.

## 5. Fibromyalgia

Fibromyalgia is defined as a neurosensory disorder characterized by widespread muscle pain, joint stiffness, and fatigue, and there are almost no inflammatory findings or abnormalities which indicate organic disorders such as bone and joint diseases, neurodegenerative diseases, rheumatic diseases, or malignant tumors. Other common symptoms of fibromyalgia present with psychosomatic characteristics, such as irritability, sleep disorders, anxiety, depression, and the onset of this disease, are seen predominantly in women in their fifties.

Fibromyalgia is reported to occur as a result of cervical spine injury in 21.6% of patients [22]. Ferrari [23] prospectively examined 268 patients and he found 2 cases (0.8%) of fibromyalgia after acute whiplash injury. Although the etiology and development processes may relate to mental fatigue or physical trauma based on genetic predisposition, the relationship between spine injury and fibromyalgia remains unclear.

## 6. Clinical Practice for Quebec Guidelines

**Figure 2:**
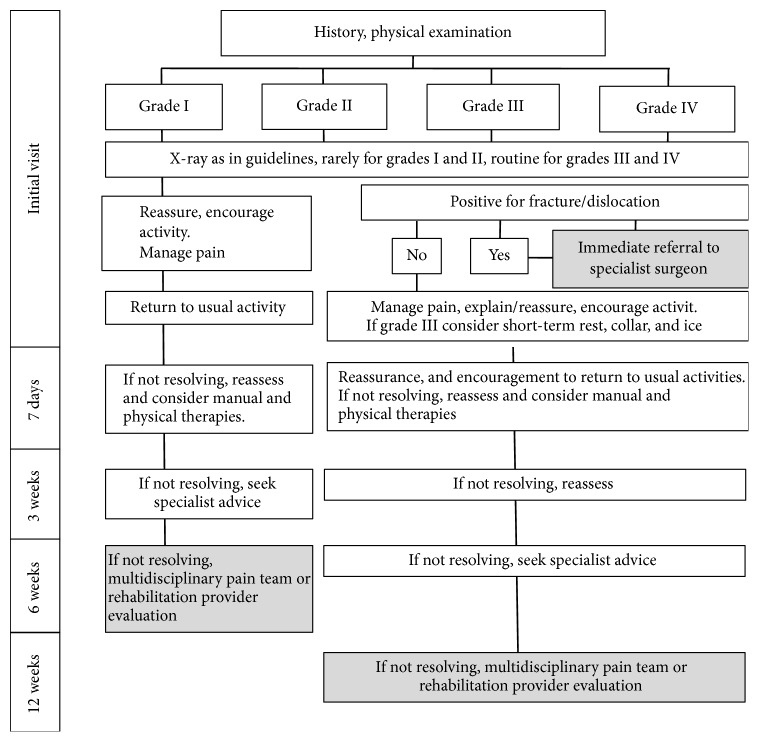
Clinical practice guidelines by Quebec Task Force on whiplash-associated disorders [2].

Clinical practice guidelines were established by the Quebec Task Force on whiplash-associated disorders [2] (Figure 2), to standardize the treatment and its duration based on the severity of symptoms. The final treatment evaluation regarding soft tissue repair is determined at 12 weeks after injury. However, as mentioned above, traumatic cervical syndrome has symptoms across diverse fields such as otolaryngology, neurology, neurosurgery, and internal medicine, as well as orthopaedic symptoms, and they overlap (functional anatomic factors), vary in duration (a time factor), and depend on the biological or social situation of the patient (individual factors). For treatment of WAD, clinicians should realise that expression of clinical symptoms is implicated in biopsychosocial model. WAD according to the Quebec classification can range from a muscle sprain to spinal cord contusions to a fractured vertebra. The latter two are rarer and can easily be detected. But, for the majority of cases, there are considerable cases who have pain with no visible cause. Therefore, the Quebec guidelines may not be applicable for all patients with traumatic cervical syndrome. Although the Quebec classification and its guidelines are very useful for standardization of the symptoms of traumatic cervical syndrome, more comprehensive guidelines are necessary to enable more accurate treatment responses to this diverse disease.

## Figures and Tables

**Table 1:** Neurological symptoms after whiplash injury [1].

Headaches	Migraine-type headache
	Tension-type headache
	Cervicogenic-type headache
	Temporomandibular joint derangement
	Greater occipital neuralgia
	Third occipital headache

Cognitive and psychological symptoms	Memory, attention, or concentration impairment
	Sleep disturbance
	Psychiatric disorders: anxiety, depression, phobic travel, anxiety, and posttraumatic stress disorder

Dizziness	Vestibular dysfunction
	Cervical origin
	Brainstem dysfunction

Visual symptoms	Blurred vision
	Reduced visual field
	Photophobia
	Disordered fusion
	Reading and driving difficulties
	Reduced accommodation

Paresthesias	Trigger points
	Brachial plexopathy
	Cervical radiculopathy
	Spinal cord compression

Weakness	Brachial plexopathy
	Cervical radiculopathy
	Spinal cord compression

Rare symptoms	Torticollis
	Tremor
	Transient global amnesia
	Hypoglossal nerve palsy
	Superior laryngeal nerve paralysis
	Cervical epidural hematoma
	Brainstem infarct
	Internal carotid and vertebral artery dissection
	Symptomatic Chiari malformation

**Table 2:** Clinical classification on whiplash-associated disorders proposed by the Quebec Task Force [2].

Grade	Clinical presentation
0	No complaint about neck pain
	No physical signs

I	Neck complaint of pain, stiffness, or tenderness
	No physical signs

II	Neck complaint
	Musculoskeletal signs including
	Decreased range of movement
	Point tenderness

II	Neck complaint
	Musculoskeletal signs
	Neurological signs including
	Decreased or absent deep tendon reflexes
	Muscle weakness
	Sensory deficits

IV	Neck complaint and fracture or dislocation

**Table 3:** Clinical spectrum of whiplash-associated disorders as proposed by the Quebec Task Force [2].

Grade	Presumed pathology	Clinical presentation

I	Microscopic or multimicroscopic lesion Lesion is not serious enough to cause muscle spasm	Usually presents to a doctor more than 24 h after trauma

II	Neck sprain and bleeding around soft tissue (articular capsules, ligaments, tendons, and muscles) Muscle spasm secondary to soft tissue injury	Usually presents to a doctor in the first 24 h after trauma
		Nonspecific radiation to the head, face, occipital region, shoulder, and arm form soft tissues injuries
		Neck pain with limited range of motion due to muscle spasm

III	Injuries to neurologic system by mechanical injury or by irritation secondary to bleeding or inflammation	Presents to a doctor usually within a few hours after the trauma
		Limited range of motion combined with neurologic symptoms and signs
